# Trainable High Resolution Melt Curve Machine Learning Classifier for Large-Scale Reliable Genotyping of Sequence Variants

**DOI:** 10.1371/journal.pone.0109094

**Published:** 2014-10-02

**Authors:** Pornpat Athamanolap, Vishwa Parekh, Stephanie I. Fraley, Vatsal Agarwal, Dong J. Shin, Michael A. Jacobs, Tza-Huei Wang, Samuel Yang

**Affiliations:** 1 Department of Biomedical Engineering, Johns Hopkins University, Baltimore, Maryland, United States of America; 2 Department of Emergency Medicine, Johns Hopkins Medicine, Baltimore, Maryland, United States of America; 3 Department of Computer Science, Johns Hopkins University, Baltimore, Maryland, United States of America; 4 Department of Mechanical Engineering, Johns Hopkins University, Baltimore, Maryland, United States of America; 5 The Russell H. Morgan Department of Radiology and Radiological Sciences, Johns Hopkins Medicine, Baltimore, Maryland, United States of America; 6 The Sidney Kimmel Comprehensive Cancer Center, Johns Hopkins Medicine, Baltimore, Maryland, United States of America; University of California, San Francisco, United States of America

## Abstract

High resolution melt (HRM) is gaining considerable popularity as a simple and robust method for genotyping sequence variants. However, accurate genotyping of an unknown sample for which a large number of possible variants may exist will require an automated HRM curve identification method capable of comparing unknowns against a large cohort of known sequence variants. Herein, we describe a new method for automated HRM curve classification based on machine learning methods and learned tolerance for reaction condition deviations. We tested this method *in silico* through multiple cross-validations using curves generated from 9 different simulated experimental conditions to classify 92 known serotypes of *Streptococcus pneumoniae* and demonstrated over 99% accuracy with 8 training curves per serotype. *In vitro* verification of the algorithm was tested using sequence variants of a cancer-related gene and demonstrated 100% accuracy with 3 training curves per sequence variant. The machine learning algorithm enabled reliable, scalable, and automated HRM genotyping analysis with broad potential clinical and epidemiological applications.

## Introduction

Nucleic acid characterization by High Resolution Melting (HRM) is a powerful technique for identifying sequence variation. By measuring the fluorescence of a saturating intercalating dye as PCR-amplified DNA fragments are heated and disassociate, sequence-defined melt curves are generated with single-nucleotide resolution in a closed-tube reaction [1], [2]. HRM curve shape and melting temperature are both related to sequence composition. But can vary slightly due to differences in the final concentration of DNA amplicon and buffer conditions. Nonetheless, its simplicity, speed, low cost, ease of use, flexibility, and high sensitivity/specificity make HRM an attractive genotyping tool with broad potential clinical diagnostic and research applications, including infectious diseases, oncology, inherited diseases, and epigenetics [3]–[16]. Also almost all modern qPCR machines include high-resolution melt functionality which is an automatic, single and direct step after target amplification. Researchers including our group have combined the use of broad-range PCR primers with HRM to enable “fingerprinting” of diverse genetic sequence heterogeneity. By amplifying three hypervariable regions within the 16S rRNA gene using flanking conserved primers followed by HRM, we were able to generate unique curve signatures from 58 bacterial species [17]. These curves were subsequently catalogued as a reference library to identify species in blinded clinical samples through curve matching. However, to enable the interrogation of larger libraries of highly polymorphic genetic loci, enhanced and automated methods to analyze HRM data for genotyping are still needed.

Current methods of curve matching rely on either arbitrary visual inspection, subtraction (difference) plot against a known control sample, or via the use of a clustering function included in the instrument software [5]–[14], [18]. Unfortunately, these methods are impractical for analysis of larger HRM data sets. A recent meta analysis of 195 studies showed that while HRM is sensitive there remains specificity issues as well as differences across instruments and analysis algorithms [19]. Given that HRM is highly sensitive to subtle variations in experimental conditions, analysis of multiple curves often requires software parameter adjustment to a low-sensitivity setting to ensure correct grouping of curves derived from identical sequences [19]. This sacrifices discriminatory power.

Some commercially available HRM analysis software such as ScreenClust [20], which is used for both variant detection [6], [7] and DNA methylation analysis [21], employs a principle component analysis (PCA) [22] to cluster all the populations together simultaneously. However, machine learning methods such as linear kernel SVM can be used to determine hyperplane on multidimensional space that optimally separates different classes [23]. For example, the optimal hyperplane maximizes the margin between vectors (i.e. melt curves) of each class. The SVM uses input (slack) parameters to determine error tolerance. The “support” vectors are created from the training data to define the hyperplane. The ability of SVM to obtain this maximum margin hyperplane makes it very powerful in the case of low noise training data, such as melt curves. Other frequently used classifying algorithms are Naive Bayes, Linear Discriminant Analysis (LDA) and k Nearest Neighbors (KNN) [24], [25]. However, we show herein that these methods are not as robust as the SVM we developed.

Herein, we adopted the use of such a machine learning algorithm based on a linear SVM to classify melt curves with trained tolerance for variations in reaction conditions. We also created an algorithm to identify the minimal set of conserved primers flanking hypervariable regions capable of discriminating all sequence variants in a given data set. As proof of concept, we demonstrated *in silico* the ability of our approach to identify all known 92 serotypes of *Streptococcus pneumoniae* based on their predicted melt curves. We further verified our method experimentally using a panel of synthetic DNA for various alleles of the human *RASSF1A* gene.

## Materials and Methods

### Primers Selection

**Figure 1:**
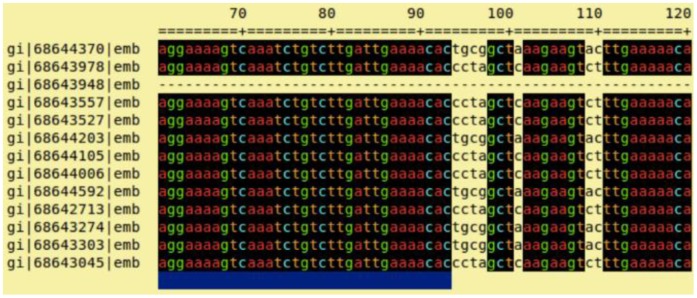
Gblocks output. The blue-highlight underneath represents the region that passes the criteria according to the parameters and this region will be considered as a candidate to be a primer.

Our primer-finding algorithm, implemented with Python, was developed to enable the selection of primer pairs among conserved regions which flank variable regions that differentiate all desired sequences. Sequences were first aligned using the multiple sequence alignment tool Kalign [26] with default parameters. Then, the aligned sequences were analyzed using Gblocks [27] to find conserved regions as shown in Figure 1. The parameters used were specific to align DNA sequences with 18 nucleotides minimum block length, no gap/no mismatch allowed, and use default values for remaining parameters. All combinations of exact-matched 18-mer from two blocks within approximately 500 base pair length were initially chosen as primers, then the regions between those primers were examined to determine how many sequences could be discriminated by each primer set using BLASTClust [28] with single nucleotide different sensitivity. BLASTClust would cluster the input DNA sequences base on the nucleotide similarity. The sequences would be grouped together if they were identical. The melting temperature, GC content of each primer site, and the number of GC differences between primers were constrained while selecting a primer pair [29]. The primer pair that could give the maximum number of distinguishable sequences was selected. A new sequence set was then created from the remaining indistinguishable sequences, and the algorithm was applied again. In this study, the capsule polysaccharide synthesis (cps) gene locus which are believed to influence the antigenic diversity in the human immune system [30] of 92 published serotypes of *S. pneumoniae* were used, including 90 serotypes from the Wellcome Trust and two recently disclosed serotypes: 6C (GenBank accession code EF538714) and 6D (accession code HM171374) [30]–[32].

### Generating Predicted Melt Curves

We generated predicted melt curves from the optimized primer sets using uMelt web application [33]. First, we used the computer algorithm to find the list of all possible amplicons that could be flanked by each primer set and then input all the amplicons into uMelt batch mode. The parameters for uMelt were set as follow: Temperature range 65°C–95°C with 0.5°C resolution and default thermodynamic set as Unified-SantaLucia 1998. We simulated data with the combination of monovalent cation [Mono+]: 47, 50, and 53 mM and [Mg++]: 1.4, 1.5, and 1.6 mM with 0% of dimethylsulfoxide for a total of 9 conditions applied to all amplicons. The output temperature and fluorescence intensity data from each sequence (Dataset S1–S7) was used for subsequent SVM analysis.

### Classification

#### Data Preprocessing

Pre-processing involves deriving a feature vector from a melt curve. Every normalized melt curve is a plot of helicity values corresponding to various temperature values, starting from helicity at 100% to helicity at 0%. We further normalize the melt curves to give us exactly 300 helicity value points between temperature values of 65 degrees and 95 degree Celsius. If the number of helicity value points generated from melt curve analysis is not 300, piecewise linear interpolation is used to ensure exactly 300 points. Since what we intend to capture is the variation in the helicity with temperature and not the exact values of helicity, we need to have a method that would be oblivious to changes in the melting points. Thus after having 300 points for each input melt curve, we rotate all the curves such that helicity values on the x axis and the temperature values become the dependent variable plotted on the y axis. This data is then interpolated to have 1000 temperature values between helicity values of 100 and 0. This acts as a feature vector input to the Machine Learning classifier. We developed our software using the Matlab programming environment (Mathworks, Natik, MA).

#### Naive Bayes based classifier

This classifier is based on Bayes theorem, which requires a large amount of estimated samples needed for accurate classification. However, Naive Bayes reduces the number of estimated parameters needed by using a conditional independence assumption. Conditional independence is defined as: if given variables A, B, and C. A and B are conditionally independent given C, if
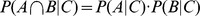



That is, if A and B are conditionally independent given C, then B contains no information about A that is not contained in C.

The Naive Bayes formulates the prior probabilities of each of the classifiers and based on the maximum likelihood estimate of the test melt curve, computes the posterior probability of the test curve belonging to each of the classes trained. We assume equal priors for each class. The class with the maximum posterior probability is assigned to the test curve.

#### k Nearest Neighbor based classifier (KNN)

**Figure 2:**
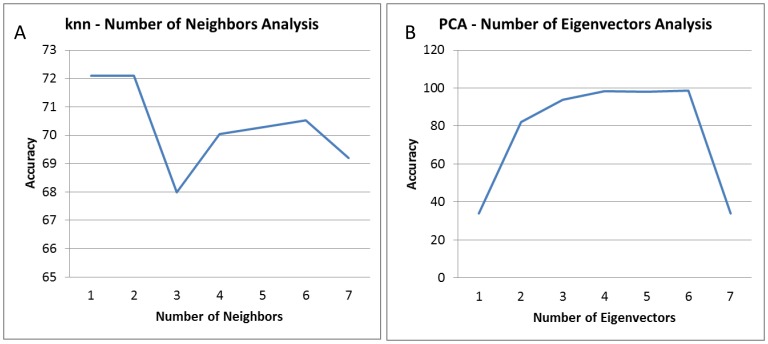
Classification results with varied parameters. A) The KNN classifiers were tested by varying number of neighbors, k from 1 to 7. The plot shows average accuracy for each k. k = 1 and k = 2 resulted in the best performance. B) PCA-LDA classification result with varied number of eigenvectors. Our PCA-LDA classifiers were tested for dimensionality reduction varied from one through seven different eigenvectors. The plot shows the highest accuracy when using six eigenvectors.

The KNN is an instance based classifier based on the similarity of each neighbor in data space using a distance metric [34]. For example, every melt curve was classified in the same class as k (pre-determined) neighboring melt curves from the training dataset based on the Euclidean distance between two data points. The number of neighboring melt curves were varied from k = 1 to k = 7 and the results show that the best performance for the classifier was k = 1. This analysis is shown in Figure 2A.

#### Principal component analysis (PCA) - Linear Discriminant Analysis (LDA) based classifier

This hybrid algorithm involves application of PCA to reduce the dimensionality of the data with subsequent application of LDA to classify the data. The LDA suffers from curse of dimensionality because the pooled covariance of the training data of melt curves was not positive semidefinite and failed. To overcome this problem, we used the PCA algorithm. PCA is a linear dimensionality reduction algorithm that maximizes the variance of lower dimensional data and was used prior to LDA. In order for LDA to run, the within class covariance matrix for the training data must be invertible i.e. full rank. Since the input data of melting curves is a sparse dataset with a large number of training points, a dimensionality reduction technique is required to reduce the number of points in training sample. Here, in this case, using PCA, we reduced the dimensionality of the training and test data and then applied the LDA on this reduced dimensional data set for classification. The PCA-LDA algorithm was tested for dimensionality reduction to one through seven different eigenvectors and the best results were achieved using six eigenvectors and shown in Figure 2B.

#### Support Vector Machine based classifier

Herein, we used a one vs. one ensemble of linear kernel SVM with Least Squares optimization. The SVM was trained with two groups of feature vectors. At each data point location, *i*, which represents a melt curve in a 1000-dimensional feature space, the melt curve was represented by a vector *x*. With this terminology we assigned a label, *y*, which uses the values of −1 and +1, to represent the melt curve type, to every possible feature vector *x*. By a statistical sampling of respective feature vectors along with their labels, the SVM method derived a detection rule by taking a pairwise similarity index between these samples *k(x,x′)* and computing the solution to the following set of equations:
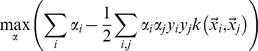



Subject to: 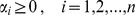


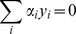



Here, the vector 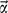 corresponds to the hyperplane such that 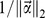 represents the margin of the hyperplane, based on the l_2_-norm. It is known that if the matrix of values *k(x,x′)* is positive semidefinite then the solution implicitly corresponds to a linear separation rule in higher dimensional space and that the distance between this linear boundary and the nearest sample points is maximized by the support feature vectors defined by constraints in the above equations [35].

Moreover, to test and validate the SVM margins, we used the leave-one-out cross validation (LOOCV) error of the detection rule:
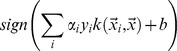



Here, b is the scalar bias term, which ensures that the hyperplane is not forced to go through the zero [23].

SVM Training: Our ensemble classifier consisted of 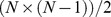 SVM feature vectors described above. Where N refers to the number of classes (e.g. 92 serotypes) the input data is grouped into. The classifier for an N class input is developed as follows: for each of the different sequences, we generated feature vectors, S_i_ where *i* = {1,2,3,…,92} as explained in the pre-processing step. Next, we trained an SVM to distinguish every S_i_ against every S_j_ for *i* = {1,2,3,…,92} and *j* = {1,2,3,…,92} giving ensemble of 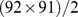 trained SVM that works as a single unit for classification.

**Figure 3:**
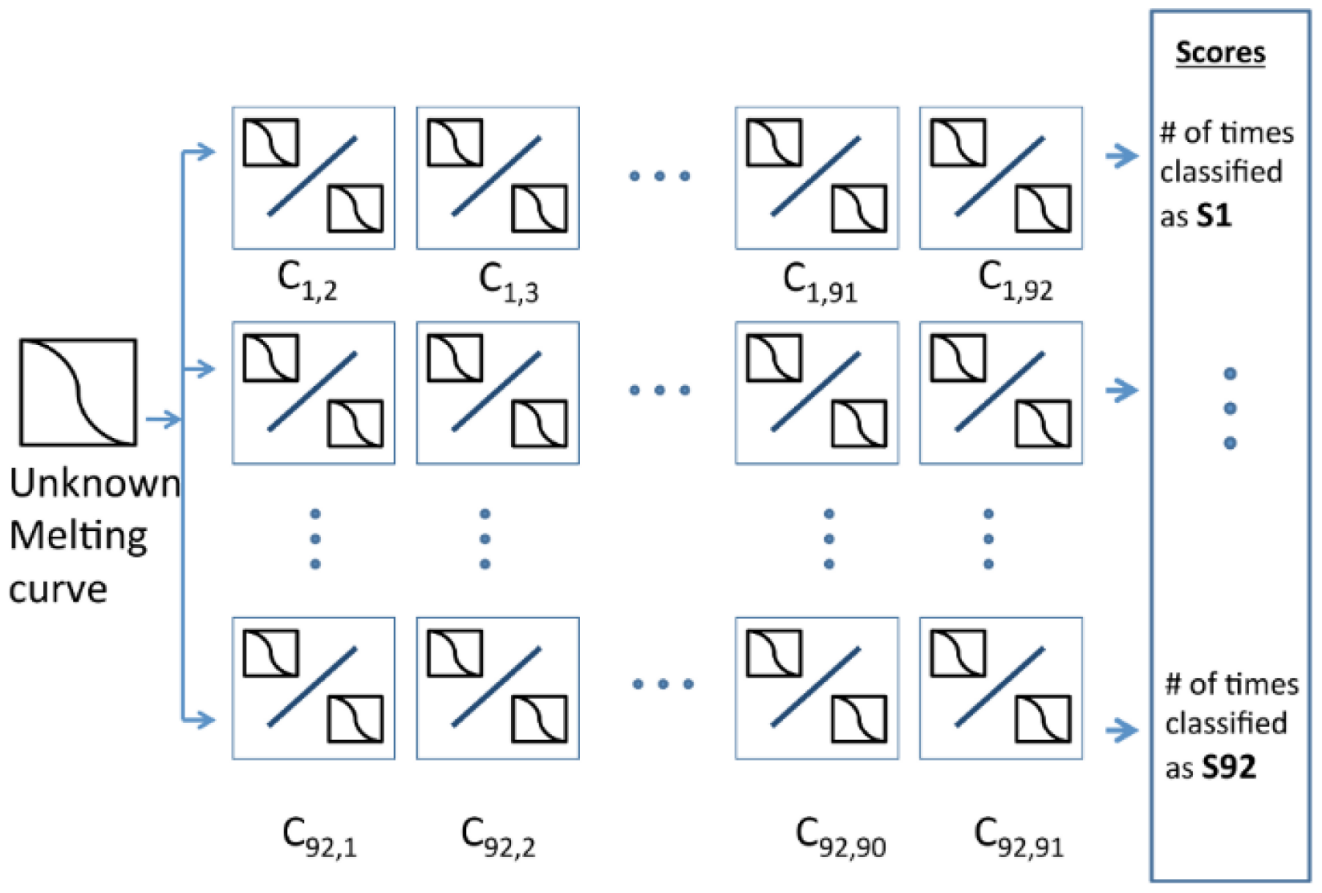
Illustration of the ensemble binary classifiers. Each classifier would be used to differentiate two classes and the score will be count for each serotype. In a SVM classifier, each class consists of 9 melt curves from 9 different conditions. The result will be based on the serotype that returns the highest score.

Decision-making: In each binary testing of the SVM, all 18 curves (9 curves from each serotype) were used. The decision making was based on the scoring scheme shown in Figure 3. Here C_i,j_ denotes the classifier that classifies *i* against *j* where *i* = {1,2,3,…,92} and *j* = {1,2,3,…,92} - i. The value of C_i,j_ is one when the curve is classified as *i* and zero when the curve is classified as j. The number of ones in every row *i* of Figure 3 indicates how many times the curve was recognized as *i*. The row with the highest score is the serotype that the melt curve is classified as. For example, let an unknown sample be 1. If the number of ones in 1st row = 87 and the number of ones in 2nd,…, 92nd row are less than 87. Then, the unknown sample would be classified as serotype 1.

### Creation of Different Melting Profiles from Synthetic DNA

Different melt curve profiles were created by using *RASS1FA* synthetic DNA. First, six 95-bp DNA templates, as shown in Table 1, were synthesized by two-step fusion PCR described by Lo et al, 2009 [36] to have 10 ‘TG’ or ‘CG’ sites at different positions. Each DNA template was created by four 35-nt primers, which have 15-nt complementary region for each primer pair. In the first round of fusion PCR, 1 µmol/L of forward and 1 µmol/L of reverse primers of each half of the sequence were annealed together in 25 µl of PCR reaction mix which contains 1.25 unit of Taq polymerase, 1X PCR buffer, and 1 mM deoxynucleoside triphosphates (dNTPs) (Invitrogen Life technologies, Carlsbad, CA). For the second round of fusion PCR, two reaction products from the first round (25 µl each) were mixed together with additional of 1.25 units of Taq polymerase added. The reaction cycle of both fusion PCRs consisted of 98°C for 1 minute, down to 60°C at the rate of −1°C/3 seconds, 60°C for 2 minutes, down to 43°C at the rate of −1°C/10 seconds, 43°C for 1 minute, up to 60°C at the rate of +1°C/ΔT sec (ΔT: the temperature difference compared to 43°C, for example, if the temperature is increasing from 51°C to 52°C, the rate will be +1°C/9 seconds [52−43 = 9], and 10 minutes for final extension at 60°C. The 20-fold diluted amplicons will be subsequently used for melt curve analysis by performing quantitative SYBR green-based PCR. The 25 µl-final volume PCR reaction contains 2 µl of the diluted amplicon, 1X Advanced SYBR Green Supermix (2X stock, Bio-Rad), and 400 nmol/L of each forward and reverse primers, which are 35-nt at the beginning and the end of each sequence respectively. The PCR program consisted of 95°C for 2 minutes, followed by 40 cycles of 95°C for 15 seconds, 60°C for 15 seconds, and 72°C for 45 seconds with another cycle for the melting step: 95°C for 15 seconds and ramping from 60°C to 95°C with ramping rate 0.2°C/sec. The melting profiles were obtained from a Bio-Rad iCycler real-time PCR machine after endpoint PCR product detection. To compensate slight well-to-well variations across the plate, we utilized the fluorescent level outside the amplicon melting region to calculate background for each well separately. Then, we performed exponential background subtraction and normalization the melt curves between 0% and 100% according to the method published elsewhere [37].

**Table 1:** List of target DNA sequences.

Seq No.	%Methylation	# of ‘CG’	Nucleotide sequence
1	100	10	5′GGGTTCGTTTTGTGGTTTCGTTCGGTTCGCGTTTGTTAGCGTTTAAAGTTAGCGAAGTACGGGTTTAATCGGGTTATGTCGGGGGAGTTTGAGTT-3′
2	80	8	5′GGGTTCGTTTTGTGGTTTCGTTCGGTTCGTGTTTGTTAGTGTTTAAAGTTAGCGAAGTACGGGTTTAATCGGGTTATGTCGGGGGAGTTTGAGTT-3′
3	60	6	5′GGGTTCGTTTTGTGGTTTCGTTTGGTTTGTGTTTGTTAGTGTTTAAAGTTAGCGAAGTACGGGTTTAATCGGGTTATGTCGGGGGAGTTTGAGTT-3′
4	40	4	5′GGGTTCGTTTTGTGGTTTCGTTTGGTTTGTGTTTGTTAGTGTTTAAAGTTAGTGAAGTATGGGTTTAATCGGGTTATGTCGGGGGAGTTTGAGTT-3′
5	20	2	5′GGGTTCGTTTTGTGGTTTCGTTTGGTTTGTGTTTGTTAGTGTTTAAAGTTAGTGAAGTATGGGTTTAATTGGGTTATGTTGGGGGAGTTTGAGT-3′
6	0	0	5′GGGTTTGTTTTGTGGTTTTGTTTGGTTTGTGTTTGTTAGTGTTTAAAGTTAGTGAAGTATGGGTTTAATTGGGTTATGTTGGGGGAGTTTGAGTT-3′

## Results

### Least primer-set selection for *Streptococcus pneumoniae*

To test our curve classification algorithm *in silico* for a model application, we set out to identify all the different serotypes of *S. pneumoniae* by PCR amplifying the capsule polysaccharide synthesis (cps) gene locus for subsequent HRM analysis. Epidemiologic surveillance of pneumococcal serotype distribution is important for assessing vaccine effectiveness and monitoring emergence of non-vaccine strains [38]. Due to PCR constraint [29], we sought for the minimum set of conserved primer pairs capable of amplifying all 92 known serotypes of *S. pneumonia*e, with each primer pair flanking regions of high sequence variability for serotype discrimination. Since none of the existing primer-finding programs available take into consideration our primer design constraints, we developed our own primer selection algorithm. As a result, we identified a set of seven conserved primer pairs (Table 2) capable of discriminating of all the 92 serotypes by their amplicon sequences *in silico*.

**Table 2:** List of 7 primer pairs used to differentiate 92 serotypes of *S. pneumonia*.

Reaction No.	Forward Primer (5′-3′)	Reverse Primer (5′-3′)	Accumulative number of differentiated serotypes	Simulated Melt curves shown in
1	GCAGTTTGTTGGACTGAC	TGGTACATAGGCATCACG	41	Dataset S1
2	ATCGCTTGGGCTTTTGCG	ATAGCCGCATCAATCACG	58	Dataset S2
3	TGGGATGCTTTCTGTGTG	CGCAAGCAGCTAAAAGCA	75	Dataset S3
4	ACCCTAGCTCAAGAAGTC	ACGATGACGAGCGACTTT	84	Dataset S4
5	CCTCCATATATGCAACAGGC	CCTGCCTGCAAGTCTTGA	87	Dataset S5
6	TCGGAGCCAATGGGTTGA	GTTAGCGGCTTGAGTTTG	90	Dataset S6
7	CAGAGGATGCTCTCGTCA	GGTAGTGGATCGGGATTG	92	Dataset S7

### Generating training data: simulated melt curves

**Figure 4:**
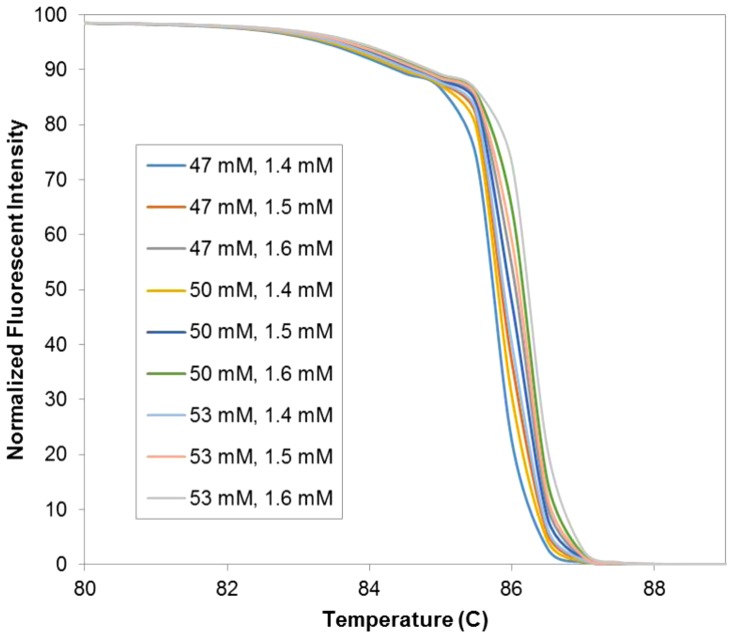
Predicted melt curves of serotype 1 with the first primer set across 9 different conditions. The predicted melt curve were generated using uMelt with 9 different conditions, which are all combinations between [Na+ K+]: 47 mM, 50 mM, and 53 mM and [Mg2+]: 1.4 mM, 1.5 mM, and 1.6 mM.

To train our melt curve classifier, amplicon sequences (Dataset S8) derived from the seven primer pairs in Table 2 were used to calculate theoretical melt curves with the web-based tool uMelt [33]. The resulting curves depend on required inputs of several PCR conditions, specifically ion concentrations, for the theoretical calculation. In order to mimic run-to-run variations in experimental conditions and considering reported intrinsic variability of 1–2% [39], [40] across different reactions and different days, we produced theoretical melt data for multiple conditions, ranging up to 5% above and below the commonly used salt concentrations (50 mM for monovalent ions such as Sodium and Potassium and 1.5 mM for Magnesium), giving us a total of 9 conditions for training our classifier. An example of simulated experimental variations in melt curves of serotype 1 derived from the first primer pair is shown in Figure 4.

### *In silico* validation of melt curve classifier for *streptococcus pneumoniae* serotyping

**Figure 5:**
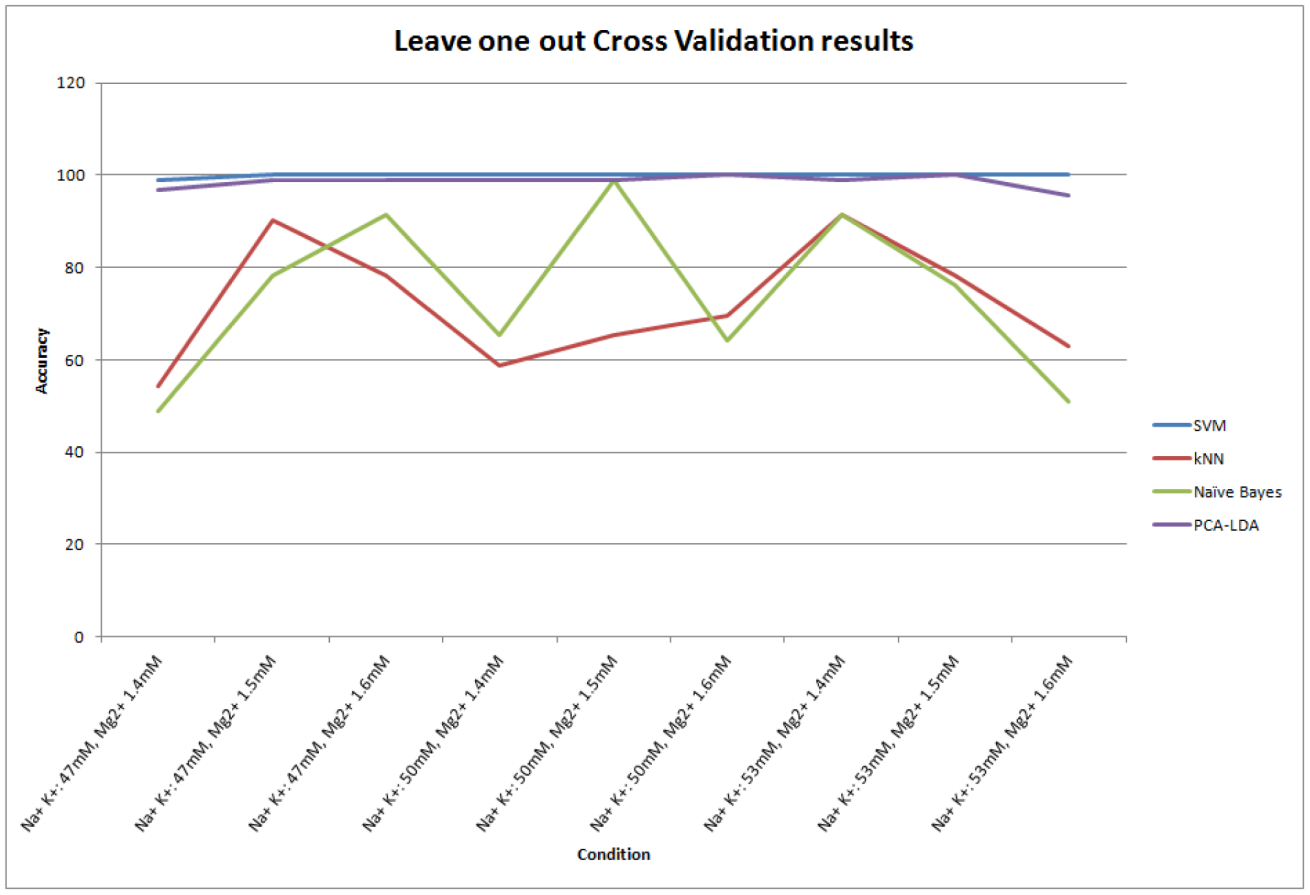
Accuracy of different classifiers under different conditions. Horizontal axis shows the different Na+, K+ and Mg2+ concentrations respectively that were used to generate the predict curves. Vertical axis shows accuracy in %age. Different curves labeled with different legends represent the performance of different classifiers.

To test the accuracy of the classifier, we performed Leave one out Cross Validation (LOOCV) which can predict the identity of a melt curve under one condition using a machine learning classifier trained on all the other remaining curves from all other conditions. We compared the results for our SVM with Naive Bayes, KNN and a newly developed PCA-LDA based classifier. We had to develop the PCA-LDA classifier because LDA alone did not work in classifying the curves. The PCA was followed by LDA to insure that the within class covariance matrix for the training data was invertible. The top six eigenvectors from the PCA results were selected for LDA classification. The KNN parameter was k = 1 for the number of neighbors as described above. Our results demonstrated that by iteratively testing each condition in this mode, the SVM based classifier resulted in an average accuracy of 99.9% as compared to 98.55% using PCA-LDA, 73.91% using Naive Bayes and the lowest 72.10% using KNN (Figure 5).

It was observed that the SVM based classifier yielded maximum accuracy. To further validate our findings, we performed two-fold (2-fold), three-fold (3-fold) and four-fold (4-fold) cross validation (CV). In general, k-fold CV involves splitting set of genotype specific melt data into k bins where the classifier is trained on data from k-1 bins and tested on the remaining bin iteratively for all possible combinations. All N-fold CV results were highly significant with an above 99.6% average accuracy. The average accuracy for each of the nine buffer conditions was defined as the mean of accuracies of all the N-fold CV tests on each buffer condition. For example, the average accuracy for condition 5 in a 3-fold CV would be obtained by averaging the accuracy CV results performed on all combinations that contain condition 5 in the test set. In case of 2-fold CV, we trained with 5 conditions and tested on the remaining 4 conditions. Detailed results for the average accuracy with 95% confidence interval for all the conditions are included in Table 3. The 95% confidence interval was calculated using the Clopper-Pearson method for calculating the exact binomial confidence interval to avoid the boundary issue [41]. Note that the accuracy drops for extreme conditions, which may be due to the fact that our model was not trained on such extremes prior to testing them. Interestingly, we found that training on only one condition and testing on the rest of the condition gave a much lower accuracy (41.4%) compared to the high accuracy (99.9%) from using all eight conditions. We also tested the classifier on newer conditions, i.e. other than original 9 conditions mentioned above. To perform this, we randomly chose two conditions within the considered extremes of the data, for example, 49 mM monovalent ions (Na+ and K+), 1.6 mM Magnesium and 52 mM monovalent ions, 1.5 mM Magnesium. The SVM classifier was able to correctly predict the serotype of all 92 samples in both of these conditions. More melt curves were also generated with higher temperature resolution settings (0.25°C) and tested with the SVM classifier trained on lower temperature resolution (0.50°C) curves. In this case, 91 out of 92 samples were predicted accurately, which gives 98.9% success rate.

**Table 3:** Average accuracy of the classifier under different Na+, K+ and Mg2+ concentrations.

No.	Condition	Leave-one-out CV			2-fold CV			3-fold CV			4-fold CV		
		Accuracy	Lower CI	Upper CI	Accuracy	Lower CI	Upper CI	Accuracy	Lower CI	Upper CI	Accuracy	Lower CI	Upper CI
1	Na^+^ K^+^: 47 mM, Mg^2+^ 1.4 mM	98.91	94.09	99.97	98.78	97.69	99.44	98.45	97.89	98.89	97.90	97.47	98.28
2	Na^+^ K^+^: 47 mM, Mg^2+^ 1.5 mM	100.00	96.07	100.00	100.00	99.50	100.00	100.00	99.86	100.00	100.00	99.93	100.00
3	Na^+^ K^+^: 47 mM, Mg^2+^ 1.6 mM	100.00	96.07	100.00	100.00	99.50	100.00	100.00	99.86	100.00	99.98	99.89	100.00
4	Na^+^ K^+^: 50 mM, Mg^2+^ 1.4 mM	100.00	96.07	100.00	99.86	99.25	100.00	99.77	99.49	99.91	99.65	99.45	99.79
5	Na^+^ K^+^: 50 mM, Mg^2+^ 1.5 mM	100.00	96.07	100.00	100.00	99.50	100.00	100.00	99.86	100.00	100.00	99.93	100.00
6	Na^+^ K^+^: 50 mM, Mg^2+^ 1.6 mM	100.00	96.07	100.00	100.00	99.50	100.00	100.00	99.86	100.00	99.98	99.89	100.00
7	Na^+^ K^+^: 53 mM, Mg^2+^ 1.4 mM	100.00	96.07	100.00	100.00	99.50	100.00	100.00	99.86	100.00	100.00	99.93	100.00
8	Na^+^ K^+^: 53 mM, Mg^2+^ 1.5 mM	100.00	96.07	100.00	100.00	99.50	100.00	99.96	99.78	100.00	99.92	99.80	99.98
9	Na^+^ K^+^: 53 mM, Mg^2+^ 1.6 mM	100.00	96.07	100.00	100.00	99.50	100.00	99.92	99.72	99.99	99.79	99.62	99.89

### *In vitro* classification validation: synthetic DNA of *RASSF1A*

**Figure 6:**
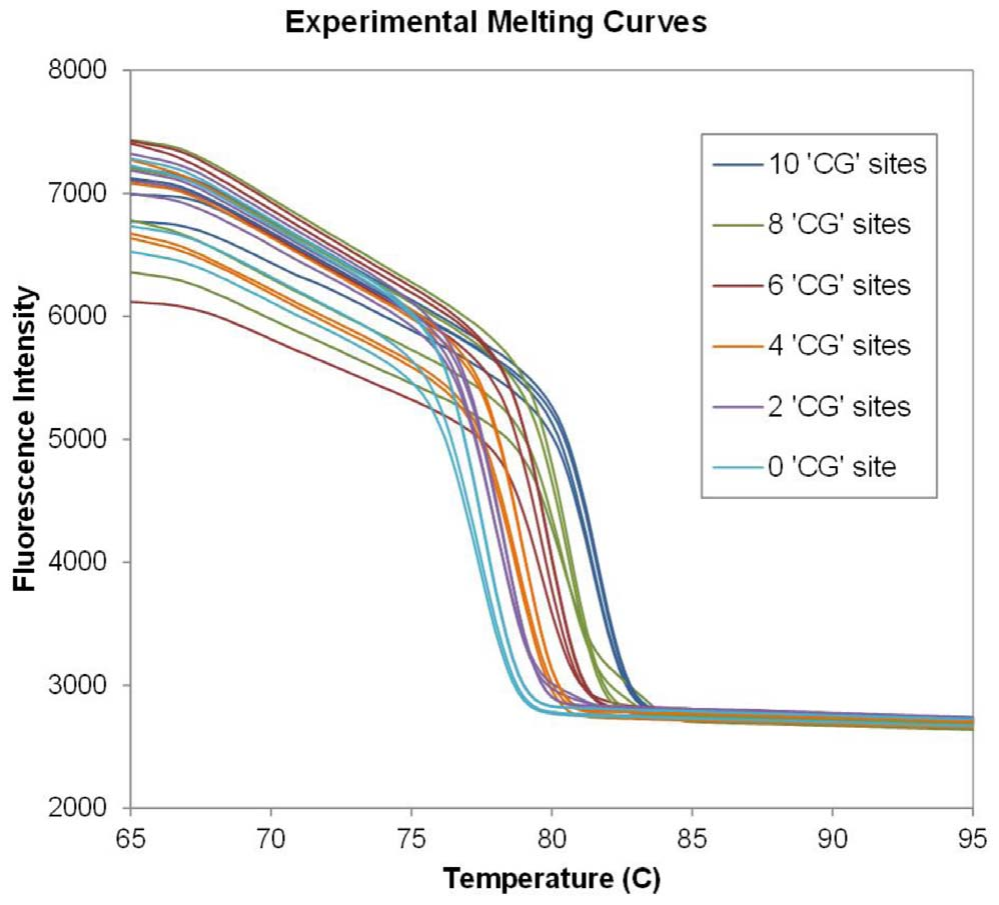
Experimental melt curves from six different number of ‘CG’ sites DNA sequences. Melt curves of six synthetic DNA sequences from two duplicate experiments from different days. Different colors represent different sequences as legend. The fully methylated sequences represented in dark blue color with 10 ‘CG’ sites and then two ‘CG’ sites were changed to ‘TG’ to be the next target of 8 ‘CG’ sites and so on until all ‘CG’ sites were changed to ‘TG’ as 0 ‘CG’ sites (non-methylated) represented in light blue.

To validate our method experimentally, we synthesized six different 95-bp DNA templates of the *RASSF1A* gene promoter sequences simulating bisulfite treated DNA containing six different methylation levels by fusion PCR, as described in a previous study [36]. Each of the sequences included different numbers of relevant ‘TG’ or ‘CG’ sites and gave a distinct melting profile. Four datasets were obtained across two duplicate experiments. Fluorescence intensities showing melt profiles of the six sequences versus temperature are plotted in Figure 6. In data preprocessing, the data was interpolated (see methods section for details) and the resolution was increased 20 fold. We used this data set to demonstrate our SVM classification model by utilizing the leave-one out cross-validation method. Only three datasets were required to train the classification model before the model could identify all six methylated genotypes with 100% accuracy.

## Discussion

We developed a novel method for broad-based classification of melt curves based on a one-versus-one ensemble SVM algorithm with a linear kernel. This enabled 97-100% identification accuracy of melt curves in the data set. The SVM outperformed three different classification methods, Naive Bayes, PCA followed by LDA and k Nearest Neighbors. Only the newly developed PCA-LDA method and SVM yielded high accuracy. However, the PCA-LDA model could be challenging since it requires a two-step procedure and the method is dependent on the eigenvectors selected from PCA to run with LDA. In addition, at 95% confidence interval, SVM (99.9, [95.85, 100]) performs better than PCA-LDA (98.55, [93.62, 100]) in LOOCV. The SVM classification model incorporates a machine learning algorithm that learns the unique characteristics of each melt curve by training multiple times with curves generated under slightly varying conditions. This training method not only enhanced the robustness and tolerance of the model against experimental variability but also increased the accuracy of identification as we concluded from k-fold cross validation test results, i.e. the more data used to train our model, the higher the accuracy achieved.

In infectious disease applications, reliable HRM curve classification would enable sequence typing of microbial organisms with considerable practical utility [17], [42]. To do so, HRM should be capable of resolving a significant number of sequence variant alleles within a genetic locus. We have developed a primer design algorithm which can generate the minimal set of PCR primers flanking hypervariable segments needed to discriminate all the input sequences of a target gene. It takes into account multiple amplicons which give multiple melting sites and optimal amplicon lengths to enhance the discriminatory power. Subsequent SVM-based analysis of unknown curves derived from these primer sets against a large data set of known controls would then allow for sequence, or microbial, identification. We demonstrated *in silico* the potential for genetic serotyping of *Streptococcus pneumonia* based melt curves. Using 7 primer pairs, we can achieve 99.9% accuracy in serotype identification based on predicted melt curves. Compared to the Quellung method, which is an antibody-based biochemical reaction against the bacterial capsule currently used as laboratory gold standard for *S. pneumoniae* serotyping, our approach provides an opportunity for a more simple, rapid, and cost-effective analysis. Alternatively, the next-generation sequencing would be a sensitive and specific detection method but it comes with errors in base calling, sequence alignment and assembly of sequence data [43]. It is time-consuming and involves multi-step process.

We also demonstrated experimentally that our approach could be used for epigenetic research applications. Typically, epigenetic analysis of DNA methylation patterns uses sodium bisulfite treatment to convert unmethylated cytosines to uracils while methylated cytosines remain unchanged. This leads to differences in sequence GC content and thus different melting profiles after PCR amplification. We experimentally validated our sequence classification method by using synthetic *RASSF1A* promoter sequences simulating six different methylation levels, which our SVM could automatically identify with 100% accuracy in the presence of both inter-assay and intra-assay variations.

Some foreseeable limitations of this method exist. For example, here we only tested homogeneous samples, but the ensemble melt curve of a mixed population could be challenging to resolve. However, we have demonstrated previously that digital PCR [44]–[47] integrated with HRM [48] allows for the separation of individual target DNA from heterogeneous samples by diluting them across many reactions. This generates individually identifiable melt curves for each genotype present. To further improve the accuracy of the training model, we anticipate that including temperature calibrator probes with additional curve normalization would be beneficial [48], [49]. Likewise, in addition to reporting the final scores from multiple classifiers, the algorithm can incorporate classification scores/confidences from each binary classifier to enhance the model decision efficacy. Also, the primer finding algorithm can be further developed to enable the user to purposefully engineer groups of identical or distinguishable melt curves according to their specific detection needs.

We have introduced a novel approach for HRM curve identification using SVM to enable highly accurate and automated identification of melt curves based on comparison to an extensive reference library. By allowing the model to learn the unique shape of each melt curve, subtle experimental variations were tolerated without loss in discrimination accuracy. As a result, our method provides a powerful tool with broad applicability in microbiology, epigenetics, as well as other types of HRM studies.

## Supporting Information

Dataset S1 **DNA sequences of amplicons and simulated melt curves from primer pair no. 1 showing in** **Table 2** **.**
(XLSX)

Dataset S2 **DNA sequences of amplicons and simulated melt curves from primer pair no. 2 showing in** **Table 2** **.**
(XLSX)

Dataset S3 **DNA sequences of amplicons and simulated melt curves from primer pair no. 3 showing in** **Table 2** **.**
(XLSX)

Dataset S4 **DNA sequences of amplicons and simulated melt curves from primer pair no. 4 showing in** **Table 2** **.**
(XLSX)

Dataset S5 **DNA sequences of amplicons and simulated melt curves from primer pair no. 5 showing in** **Table 2** **.**
(XLSX)

Dataset S6 **DNA sequences of amplicons and simulated melt curves from primer pair no. 6 showing in** **Table 2** **.**
(XLSX)

Dataset S7 **DNA sequences of amplicons and simulated melt curves from primer pair no. 7 showing in** **Table 2** **.**
(XLSX)

Dataset S8 **DNA sequences of amplicons from seven primer pairs.**
(XLSX)
